# The effect of osteopathic manipulative treatment on lenght of stay and pain relief in pediatric appendectomy: a pilot non-randomized time-controlled clinical trial

**DOI:** 10.3389/fped.2025.1579645

**Published:** 2025-08-04

**Authors:** Roberto Lo Piccolo, Michele Maria Cantagalli, Tommaso Ferroni, Florinda Fracchiolla, Antonino Morabito

**Affiliations:** ^1^ Department of Neurosciences, Psychology, Drug Research and Child Health (NEUROFARBA), University of Florence, Florence, Italy; ^2^ Department of Pediatric Surgery, Meyer Children’s Hospital, Florence, Italy; ^3^ Osteopathy, Meyer Children’s Hospital IRCCS, Florencer, Italy

**Keywords:** pediatric surgery, osteopathic treatment of children, fast recovery, pain management, appendicectomy

## Abstract

**Introduction:**

Appendicitis is the most frequent non-traumatic surgical emergency in children. While laparoscopic surgery is standard, postoperative recovery often involves pain, delayed bowel function, and reduced mobility. Osteopathic manipulative treatment (OMT) may improve recovery by addressing fascial restrictions and visceral dysfunction. This pilot study investigates OMT's effect on postoperative pain and hospital length of stay in pediatric patients undergoing appendectomy.

**Methods:**

This non-randomized, time-controlled clinical trial was conducted at Meyer Pediatric Hospital (Florence, Italy) with 43 patients aged 5–17 undergoing laparoscopic appendectomy. Participants were divided by appendicitis type (complicated/uncomplicated) and treatment group (OMT vs. control). The OMT group received two standardized sessions within 48 h post-surgery. Primary outcomes included postoperative pain (assessed via Numeric Rating Scale) and hospital stay. Secondary outcomes included bowel function, mobilization, and nausea/vomiting. Data were analyzed using multivariate statistics and *t*-tests, with *p* < 0.05 as the significance threshold.

**Results:**

The OMT group showed a shorter mean hospital stay (4.6 vs. 7 days) and significantly greater reductions in abdominal and shoulder pain compared to controls. In uncomplicated appendicitis, pain reduction reached 3/10 vs. 1.7/10 in controls; in complicated cases, 3.6/10 vs. 1.8/10. Shoulder pain relief was also more pronounced in the OMT groups. Improvements in bowel function, mobilization, and nausea were observed in both groups, with no statistically significant differences.

**Conclusions:**

This pilot study provides preliminary evidence that OMT may enhance postoperative recovery in pediatric appendectomy by reducing pain and potentially shortening hospital stays. Although not statistically significant due to the small sample size, the clinical relevance of these findings supports further investigation through larger, randomized trials.

## Introduction

Appendicitis, a prevalent ailment, and the foremost cause of acute abdomen (1), exhibits a distinct prevalence in pediatric populations, particularly in infancy, adolescence, and among males (2). Although individuals of all ages can be affected, pediatric cases, constituting the most common non-traumatic pediatric emergency, are predominant in children over 2 years of age. The incidence is 0.4 below 14 years, with a male-to-female ratio of 3:2 in children and adolescents, evening out to an equal ratio after 25 years (3).

Early diagnosis is pivotal for effective treatment, as a diagnostic delay exceeding 36 h increases the probability of perforation by 65%. Perforated appendicitis is predominantly observed in children under 2 years (95%), ranging from 80% to 100% in 2–5-year-olds to 10%–20% in 10–17-year-olds (3, 4).

The diagnostic approach relies mainly on clinical evaluation, emphasizing a thorough patient history and objective examination. Untreated, the perforation complication escalates mortality from 0.1% to over 5% (1).

Surgical treatment involves two approaches: open access (OA) and laparoscopic (VLA). While proponents advocate for the advantages of minimally invasive methods (VLA) in terms of faster, less painful postoperative recovery, fewer complications, and improved cosmesis, others argue for certain benefits of the open technique, such as lower postoperative abscess rates and reduced costs (1). The use of VLA significantly reduces wound infections, achieved through routine protection during organ extraction. However, non-use of protection (e.g., in laparo-assisted techniques with a single trocar) results in infection rates comparable to OA (5, 6).

A complete peritoneal cavity washout is recommended in cases of peritonitis. Routine drainage is not indicated, but it may be therapeutic in the presence of abscesses or prophylactic in specific high-risk situations (steroid therapy, chronic diseases) in selected patients. The routine use of drainage is neither necessary nor may be harmful, as suggested by a comprehensive meta-analysis. However, it is accepted in cases of diffuse peritonitis or abscesses (7).

Surgery-related complications may include deep intrabdominal abscesses, observed in 5%–10% of perforated appendicitis cases. Conservative treatment with antibiotic therapy is recommended. Prolonged paralytic ileus or intestinal obstruction, occurring in 3%–5%, is managed conservatively with fasting, nasogastric tube, electrolyte, and protein compensation (8). Rare complications include Stump Appendicitis and fecal fistulas.

In recent years, the focus has shifted to post-surgical considerations, encompassing local alterations in abdominal mobility, skin, fasciae, and scar outcomes, as well as systemic symptoms and connections (8).

Beyond surgical techniques, growing attention has been directed toward post-operative recovery, particularly in terms of functional, fascial, and systemic consequences (9, 10). Postoperative physiological changes can extend beyond the local tissue level, affecting the fascial system, visceral mobility, and neurovegetative balance, and may contribute to symptoms such as chronic pain, bloating, or postural alterations (11–15). These effects are increasingly understood within the framework of the interoceptive paradigm, which conceptualizes health and disease in terms of altered interoception—defined as the processing of internal bodily signals—and its role in modulating the autonomic nervous system and central sensitization (16, 17, 19).

In the postoperative setting, three key components often interplay: inflammation and altered interoception; peripheral and central sensitization (e.g., hyperalgesia, allodynia); and dysregulation of the autonomic nervous system, impacting homeostasis and allostasis (18). This framework provides a compelling rationale for integrating osteopathic manipulative treatment (OMT) into post-surgical care. OMT, a manual therapy rooted in the fundamental osteopathic principles that the body is a unit, structure and function are reciprocally interrelated, the body possesses self-regulatory mechanisms, and rational treatment is based on these principles, may restore functional balance by addressing somatic dysfunction and modulating neural, circulatory, and fascial pathways (19–21). Several studies have demonstrated that OMT can positively influence both interoceptive processing and autonomic regulation (19–23), offering a plausible mechanism for its benefits in enhancing recovery.

OMT is a form of manual therapy developed by Andrew Taylor Still in the late 19th century, based on the principle that the body possesses self-regulatory mechanisms to maintain health and recover from disease (24). It focuses on the interrelationship between structure and function, utilizing various manual techniques to improve mobility, circulation, and nervous system regulation.

A key concept in osteopathy is somatic dysfunction, which refers to impaired or altered function of the musculoskeletal system and its impact on overall physiological processes. This condition is recognized in the International Classification of Diseases (ICD-11) (25).

Globally, OMT has been integrated into diverse healthcare systems, with research supporting its efficacy in various conditions, including musculoskeletal pain, neurological disorders, and postoperative recovery. In Italy, osteopathy was officially recognized as a healthcare profession in 2021 through Law No. 3/2018 and Law No. 145/2018, highlighting its growing role in the national healthcare landscape (26, 27).

Although research on OMT's efficacy in postoperative care has mainly focused on adults—showing improvements in gastrointestinal motility, pain control, and reduced hospital stays—limited evidence exists in the pediatric population (28–31). The inflammatory and metabolic load induced by surgical intervention, especially in abdominal procedures, underscores the need for strategies that support physiological resilience and functional recovery. From this perspective, OMT may represent a non-pharmacological, low-risk adjunctive therapy with potential to reduce hospitalization time and mitigate post-surgical symptoms.

The pediatric domain remains largely unexplored in this context, representing a gray area in need of further investigation. This study investigates the potential benefits of OMT in pediatric patients undergoing appendectomy, assessing its impact on hospital length of stay and postoperative pain relief. The primary aim is to evaluate whether OMT reduces postoperative pain and accelerates recovery. Secondary aims include assessing improvements in mobilization, bowel function, and nausea/vomiting reduction.

## Materials and methods

### Study design

This is a pilot, non-randomized, time-controlled clinical trial conducted at Meyer Pediatric Hospital, Florence, Italy, between October 1, 2020, and September 30, 2021. The study was approved by the hospital's Ethics Committee (Protocol No. 004545), and informed consent was obtained from all participants' legal guardians.

### Participants

Patients aged 5–17 years undergoing appendectomy for acute appendicitis were eligible. Exclusion criteria included severe comorbidities (neurological, rheumatological, or musculoskeletal conditions) and prophylactic appendectomy during other abdominal interventions.

### Grouping and intervention

Patients were divided into four groups based on appendicitis type and whether they received OMT (Table 1):

**Table 1:** Patients grouping.

Group	Appendicitis type	OMT status
OMT-Uncom	Uncomplicated	OMT received
OMT-Com	Complicated	OMT received
CG-Uncom	Uncomplicated	No OMT (Control)
CG-Com	Complicated	No OMT (Control)

The OMT group received two standardized treatment sessions within 48 h postoperatively: the first (T0) within 24 h and the second (T1) within 48 h. Based on the classification proposed by Tramontano (33) this approach qualifies as standardized, as the techniques and anatomical targets were predefined rather than tailored dynamically to each patient. All participants received the same protocol, which included myofascial release, visceral mobilization, and lymphatic drainage, aimed at reducing fascial restrictions, addressing potential adhesions, and enhancing diaphragmatic and visceral mobility (32).

OMT was delivered by clinicians from Meyer Pediatric Hospital, who held a diploma in osteopathy and had formal education and certification in osteopathic practice, involving a minimum of 5 years of training and at least 1,500 h of classroom and practical training. As employed professionals of the hospital, they performed the treatment autonomously, without supervision, ensuring consistent application by trained practitioners.

### Outcomes and data collection

Primary outcomes included:

• Pain intensity assessed using the validated Visual Analog Scale (VAS) (34).
• Length of hospital stay.

Secondary outcomes included:

• Mobilization (scale: in bed, room, ward, outside ward, stairs).
• Bowel function (scale: non-canalized, gas, feces, stimulated bowel movement).
• Nausea/Vomiting (scale: none, nausea, vomiting).

Outcome assessments were performed pre- and post-treatment at T0 and T1. Data was analyzed by an independent statistician.

### Statistical analysis

Data was analyzed using SPSS software, employing multivariate analyses and linear regressions, combined with studies on statistical relationships and dispersion, and ultimately utilizing the *t*-test to assess the statistical significance of results. A *p*-value <0.05 was considered statistically significant.

## Results

The database collected was formed by 43 patients, divided into 19 patients with osteopathic treatment and 24 patients forming the control group. A total of 23 patients were excluded from the study due to the previously mentioned exclusion criteria.

**Figure 1:**
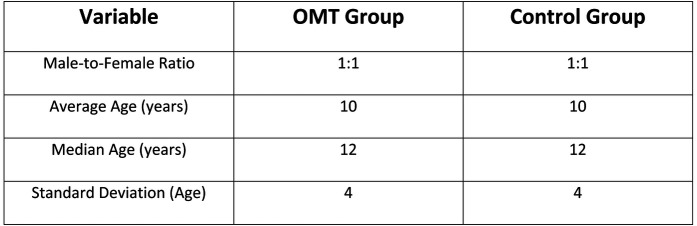
Baseline table.

In the analyzed cohort, the gender distribution was balanced, with a male-to-female ratio of 1:1. The average age at the time of surgery was 10 years, with a median age of 12 years and a standard deviation of 4 (Figure 1).

The most significant outcome was the average length of hospital stay: patients receiving osteopathic treatment (OMT) had an average stay of 4.6 days, compared to 7 days in the control group. This reduction not only reflects a form of tertiary prevention—minimizing complications and supporting functional recovery in line with the professional competencies of Italian osteopaths—but also has important economic implications by decreasing hospitalization costs for the healthcare system.

While there exists a disparity in the mean duration of hospitalization between the two groups, it fails to attain statistical significance, attributed to the limited sample size, yielding a *t*-test value of 0.19. This implies that our confidence interval will not achieve the conventional 95% level but instead rests at 80%.

**Figure 2:**
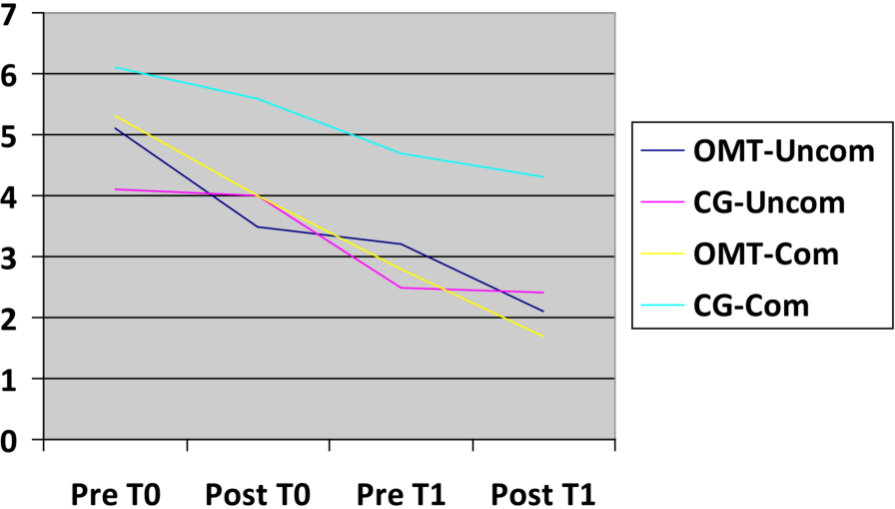
Abdominal pain relief trend. X-axis: time related to treatment (T0,T1); Y-axis: NRS scale.

Within OMT-Uncom group, the assessment of pain at T0 and T1 revealed a notable decrease in abdominal pain after osteopathic intervention, representing an average difference of 1.6/10 on the Numeric Rating Scale (NRS). A discernible, albeit less pronounced reduction persisted on the second osteopathic treatment (averaging 1.1/10 on the NRS scale). In CG-Uncom group the reduction in pain was almost absent in both first and second postoperative day (average difference of 0.1/10 on the NRS scale). When comparing the first and last pain assessments, OMT-Uncom group demonstrated an average reduction of 3/10 on the NRS scale, almost doubling the reduction observed in CG-Uncom, which averaged 1.7/10 on the NRS scale (Figure 2).

Similar observations apply to the other two comparable groups, OMT-Com and D. In OMT-Com group, there was a reduction in abdominal pain by 1.3/10 on the NRS scale following the initial osteopathic treatment, with a subsequent decrease of 1.1/10 after the second osteopathic intervention, resulting in a cumulative reduction in abdominal pain of 3.6/10 on the NRS scale before and after the two osteopathic treatments. Conversely, in CG-Com group, the reduction in abdominal pain was less pronounced, registering 0.5/10 after the first postoperative day and 0.4/10 on the second postoperative day, indicating an overall decrease in abdominal pain of 1.8/10 on the NRS scale (Figure 2).

Regarding relief from shoulder pain, OMT-Uncom exhibited a reduction of 0.6/10 on the NRS scale for pain in the left shoulder following the first osteopathic treatment and a further reduction of 0.9/10 after the second treatment, indicating an overall reduction in left shoulder pain on the NRS scale of 1.7/10 in the first 48 h after the surgery. In CG-Uncom group there was a trascurable reduction in the first 48 h post-surgery (0.2/10 on the NRS scale).

In OMT-Com and CG-Com groups, the overall reduction in right shoulder pain after the two osteopathic treatments was 2.5/10 for OMT-Com group, whereas CG-Com group experienced an increase of 0.3/10 in left shoulder pain within the first 48 h.

Investigating referred pain to the contralateral shoulder, namely the right shoulder, the overall reduction in pain was 2.3/10 on the NRS scale for OMT-Uncom group, compared to a reduction of 0.2/10 for CG-Uncom. Regarding patients with complicated appendicitis, the reduction in referred pain to the right shoulder was 2.5/10 for OMT-Com group, contrasting with an increase of 0.3/10 on the NRS scale for CG-Com.

**Figure 3:**
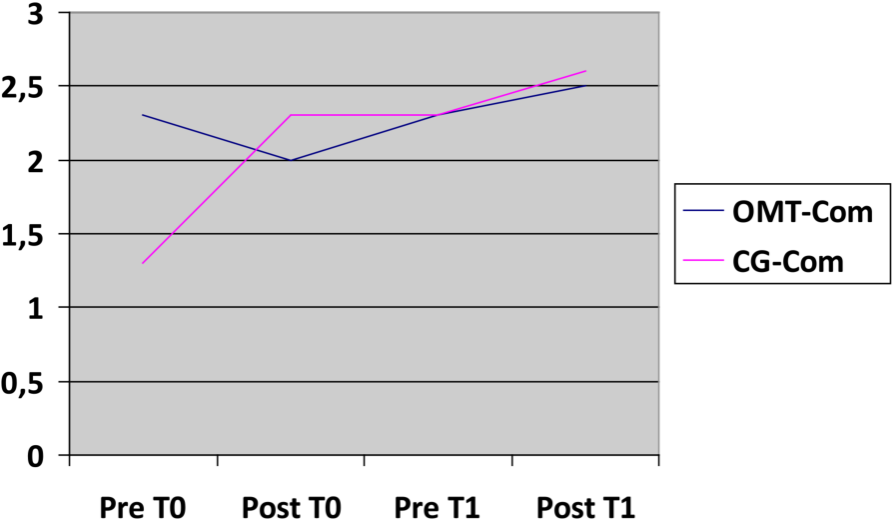
Bowel improvement. X-axis: time related to treatment (T0,T1); Y-axis: 1—no bowel movement, 2—gas, 3—fecis.

The statistical analysis on bowel improvement following two osteopathic treatments demonstrated a 1.2/3 improvement in OMT-Uncom compared to a 0.8/3 improvement in the first 48 postoperative hours in CG-Uncom group. In the case of patients with complicated appendicitis, there was a 0.2/3 improvement in bowel function in OMT-Com, as opposed to a 1.3/3 improvement in CG-Com group. This latter analysis might suggest a greater improvement in bowel function for the CG-Com compared to OMT-Com. However, this is explained by the fact that the final average values assigned to our patients, based on the bowel scales previously outlined, are indeed identical in both groups, i.e., 2.6 out of a maximum of 3 points achievable. To illustrate this concept, a graph has been constructed (Figure 3) to show that, although the Control Group seemingly achieved greater improvement, the result is identical in both groups and tends toward the maximum achievable score according to the predetermined scales.

**Figure 4:**
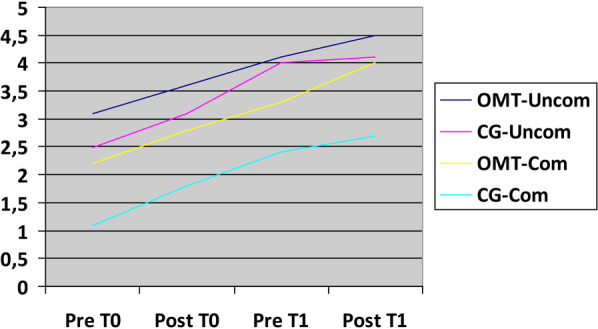
Mobilization improvement. X-axis time related to treatment (T0,T1); Y-axis: 1—absent, 2—in the bed, 3—n the room, 4—in the ward, 5—outside the ward, 6—stairs.

**Figure 5:**
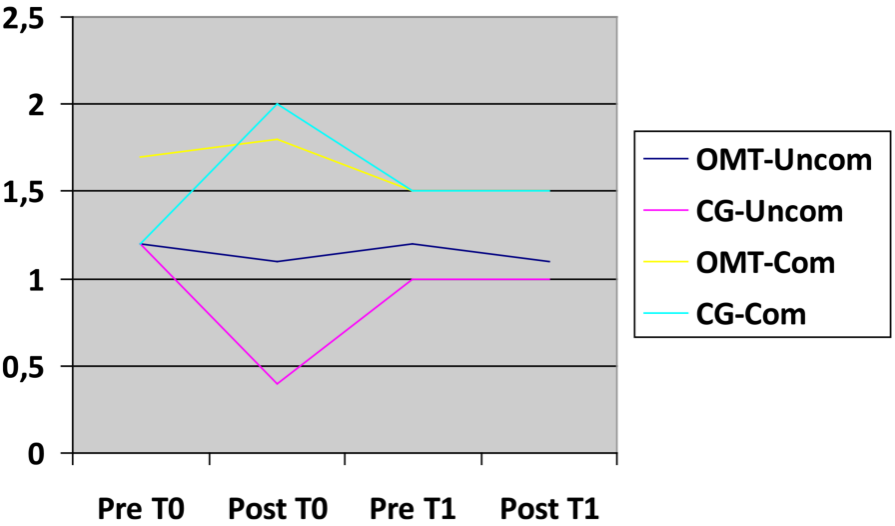
Nausea/vomiting improvement. X-axis: time related to treatment (T0,T1); Y-axis: 0—absent, 1—nausea, 2—vomiting, 3—use of nasogastric tube.

**Figure 6:**
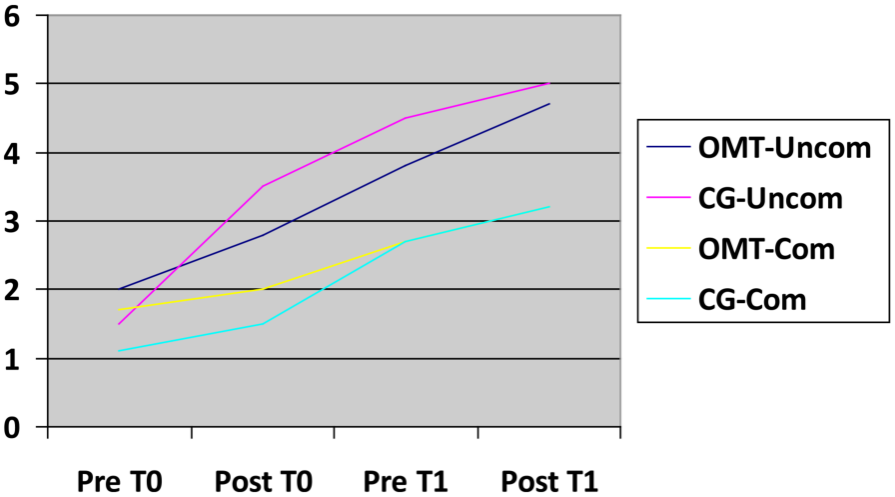
Refeeding improvement. X-axis: time related to treatment (T0,T1); Y-axis: 1—fasting, 2—water only, 3—clear liquid diet, 4—full liquid diet, 5—bland diet, 6—regular diet.

The results about mobilization, refeeding and nausea/vomiting improvement are presented below as graphs depicting the mean values across the various groups under analysis (Figures 4–6).

## Discussion

This study represents the first investigation into the role of OMT in supporting postoperative recovery in pediatric patients undergoing appendectomy, marking a novel and relevant contribution to both osteopathic research and pediatric surgical care. The findings provide promising evidence of OMT's potential to alleviate postoperative pain and reduce hospital length of stay—outcomes achieved in the absence of any recorded side effects. These results are particularly significant given the increasing emphasis on non-pharmacological, integrative strategies that promote functional recovery in hospitalized patients.

The observed benefits may be interpreted through the lens of the interoceptive paradigm (17), which frames health and disease in terms of altered interoception, central sensitization, and autonomic dysregulation. Surgical procedures, especially abdominal ones, represent a substantial inflammatory and metabolic burden, potentially leading to dysfunction across these domains. OMT, by acting on somatic and visceral structures, may help modulate interoceptive signaling and autonomic tone, thereby enhancing the body's capacity for recovery. Prior studies have demonstrated OMT's influence on heart rate variability and central sensitization markers, lending theoretical support to the mechanisms proposed here (18–20).

### Reduction in hospital stay: A clinically, preventively, and economically relevant outcome

One of the most notable outcomes of this study was the reduction in hospital stay: patients receiving OMT had an average length of stay of 4.6 days compared to 7 days in the control group. While the difference did not reach statistical significance, likely due to the limited sample size, the 2.4-day reduction is clinically meaningful. This result represents a form of tertiary prevention, as it may prevent complications and accelerate recovery—an approach consistent with the professional competencies of Italian osteopaths. Moreover, such a reduction has important economic implications, as shorter hospital stays are associated with lower healthcare costs and resource use. These findings align with evidence from Lanaro et al. (35), who showed that OMT reduced hospitalization time and associated costs in preterm infants, highlighting OMT's cost-effectiveness in other vulnerable populations.

### Secondary recovery parameters: postoperative pain reduction through interoceptive modulation and shoulder pain and referred pain mechanisms

OMT proved effective in reducing postoperative abdominal pain, particularly in patients with uncomplicated appendicitis. The reduction observed in the OMT group was consistently greater compared to the control group, indicating the potential of OMT to enhance pain management following surgery. Similar trends were noted in cases of complicated appendicitis, with OMT again leading to more pronounced pain relief. These findings align with previous research on OMT's benefits in postoperative settings, particularly in adults, where improvements in pain control and gastrointestinal function have been documented (22–24). Physiologically, this effect may be linked to OMT's ability to modulate viscerosomatic reflexes, improve tissue interoception, and normalize altered fascial tensions.

A distinctive aspect of this study was the focus on shoulder pain, a common postoperative complaint following laparoscopy, often attributed to diaphragmatic irritation. The results indicate that OMT effectively reduced shoulder pain, both in uncomplicated and complicated appendicitis cases, contrasting with the limited pain relief observed in the control groups. This improvement may be attributed to enhanced fascial mobility and the resolution of somatic dysfunctions within the thoracoabdominal area, consistent with osteopathic models that emphasize fascial continuity and segmental integration (19–21).

### Strengths, limitations and future directions

This study has several strengths: it was conducted in a real clinical scenario, ensuring high external validity; it addressed a novel topic—the integration of osteopathy in pediatric postoperative care—filling an existing gap in the literature; and it employed a well-defined, replicable treatment protocol delivered by certified professionals. Although the findings offer promising insights, several limitations must be acknowledged. First, the relatively small sample size limits the statistical power of the study, suggesting the need for larger, multicenter trials to validate the results. Second, the lack of long-term follow-up prevents conclusions about the persistence of short-term benefits over time; future research should investigate the durability of these effects. Finally, the manual nature of the OMT made double-blinding impractical, which may introduce bias despite the methodological precautions taken.

## Conclusions

This study demonstrates the potential benefits of osteopathy in reducing hospital stay and postoperative pain following appendectomy. The observed reduction in abdominal and shoulder pain, along with a clinically significant decrease in hospitalization duration, suggests that OMT could be a valuable adjunct therapy in postoperative recovery. While further studies with larger sample sizes are warranted, these findings contribute to the growing body of evidence supporting the integration of osteopathic techniques into postoperative care protocols.

## Data Availability

The raw data supporting the conclusions of this article will be made available by the authors, without undue reservation.
