# Supplementary material for: The effect of osteopathic manipulative treatment on lenght of stay and pain relief in pediatric appendectomy: a pilot non-randomized time-controlled clinical trial
Source: Front Pediatr. 2025 Aug 4;13:1579645. doi: 10.3389/fped.2025.1579645 (PMC12358424; doi:10.3389/fped.2025.1579645)

Appendix 1. Questionnaire template

Nome Data intervento

Cognome Tecnica operatoria: □ VLS □ Open □ VLS convertita in Open

Data di nascita Appendicectomia in urgenza: □ Non complicata □ Complicata

Trattato n. □ Controllo n. Appendicectomia in elezione: □ DAR □ Pregresso ascesso

**1° TMO**

Pre – trattamento (data e ora_________________) Post – trattamento (data e ora___________________)

| Dolore addominale | | | | |  |  | **Febbre** | | | Sì | No |  | Dolore addominale | | | | |  |  | **Febbre** | | | Sì | No |  |
| --- | --- | --- | --- | --- | --- | --- | --- | --- | --- | --- | --- | --- | --- | --- | --- | --- | --- | --- | --- | --- | --- | --- | --- | --- | --- |
| Dolore spalla destra | | | | |  |  |  |  |  |  |  |  | Dolore spalla destra | | | | |  |  |  |  |  |  |  |  |
| Dolore spalla sinistra | | | | |  |  |  |  |  |  |  |  | Dolore spalla sinistra | | | | |  |  |  |  |  |  |  |  |
|  |  |  |  |  |  |  |  |  |  |  |  |  |  |  |  |  |  |  |  |  |  |  |  |  |  |
| **Alvo** | | | | |  |  | **Nausea o vomito** | | | | |  | **Alvo** | | | | |  |  | **Nausea o vomito** | | | | | |
| 1 | Non canalizzato | | | |  |  | 1 | No | | | |  | 1 | Non canalizzato | | | |  |  | 1 | No | | | | |
| 2 | Gas | | | |  |  | 2 | Nausea | | | |  | 2 | Gas | | | |  |  | 2 | Nausea | | | | |
| 3 | Feci | | | |  |  | 3 | Vomito | | | |  | 3 | Feci | | | |  |  | 3 | Vomito | | | | |
| 4 | Con stimolazione | | | |  |  | 4 | SNG | | | |  | 4 | Con stimolazione | | | |  |  | 4 | SNG | | | | |
|  |  |  |  |  |  |  |  |  |  |  |  |  |  |  |  |  |  |  |  |  |  |  |  |  |  |
| **Mobilizzazione** | | | | |  |  | **Rialimentazione** | | | | |  | **Mobilizzazione** | | | | |  |  | **Rialimentazione** | | | | | |
| 1 | No | | | |  |  | 1 | No | | | |  | 1 | No | | | |  |  | 1 | No | | | | |
| 2 | Sul letto | | | |  |  | 2 | Idrica | | | |  | 2 | Sul letto | | | |  |  | 2 | Idrica | | | | |
| 3 | Stanza | | | |  |  | 3 | Thè e biscotti | | | |  | 3 | Stanza | | | |  |  | 3 | Thè e biscotti | | | | |
| 4 | Reparto | | | |  |  | 4 | Semiliquida | | | |  | 4 | Reparto | | | |  |  | 4 | Semiliquida | | | | |
| 5 | Fuori reparto | | | |  |  | 5 | Leggera/Libera | | | |  | 5 | Fuori reparto | | | |  |  | 5 | Leggera/Libera | | | | |
| 6 | Scale | | | |  |  |  |  | | | |  | 6 | Scale | | | |  |  |  |  | | | |  |
|  |  | | | |  |  |  |  | | | |  |  |  | | | |  |  |  |  | | | |  |
| Note: | | | | | | | | | | | | | | | | | | | | | | | | | |

**2° TMO**

Post-trattamento (data e ora_______________) Post-trattamento (data e ora_________________)

| Dolore addominale | | | | |  |  | **Febbre** | | | Sì | No |  | Dolore addominale | | | | |  |  | Febbre | | | | Sì | No |
| --- | --- | --- | --- | --- | --- | --- | --- | --- | --- | --- | --- | --- | --- | --- | --- | --- | --- | --- | --- | --- | --- | --- | --- | --- | --- |
| Dolore spalla destra | | | | |  |  |  |  |  |  |  |  | Dolore spalla destra | | | | |  |  |  |  |  |  |  |  |
| Dolore spalla sinistra | | | | |  |  |  |  |  |  |  |  | Dolore spalla sinistra | | | | |  |  |  |  |  |  |  |  |
|  |  |  |  |  |  |  |  |  |  |  |  |  |  |  |  |  |  |  |  |  |  |  |  |  |  |
| **Alvo** | | | | |  |  | **Nausea o vomito** | | | | |  | **Alvo** | |  |  |  |  |  | **Nausea o vomito** | | | | |  |
| 1 | Non canalizzato | | | |  |  | 1 | No | | | |  | 1 | Non canalizzato | | | |  |  | 1 | No | | | |  |
| 2 | Gas | | | |  |  | 2 | Nausea | | | |  | 2 | Gas | | | |  |  | 2 | Nausea | | | |  |
| 3 | Feci | | | |  |  | 3 | Vomito | | | |  | 3 | Feci | | | |  |  | 3 | Vomito | | | |  |
| 4 | Con stimolazione | | | |  |  | 4 | SNG | | | |  | 4 | Con stimolazione | | | |  |  | 4 | SNG | | | |  |
|  |  |  |  |  |  |  |  |  |  |  |  |  |  |  |  |  |  |  |  |  |  |  |  |  |  |
| **Mobilizzazione** | | | | |  |  | **Rialimentazione** | | | | |  | **Mobilizzazione** | | | | |  |  | **Rialimentazione** | | | | |  |
| 1 | No | | | |  |  | 1 | No | | | |  | 1 | No | | | |  |  | 1 | No | | | |  |
| 2 | Sul letto | | | |  |  | 2 | Idrica | | | |  | 2 | Sul letto | | | |  |  | 2 | Idrica | | | |  |
| 3 | Stanza | | | |  |  | 3 | Thè e biscotti | | | |  | 3 | Stanza | | | |  |  | 3 | Thè e biscotti | | | |  |
| 4 | Reparto | | | |  |  | 4 | Semiliquida | | | |  | 4 | Reparto | | | |  |  | 4 | Semiliquida | | | |  |
| 5 | Fuori reparto | | | |  |  | 5 | Leggera/Libera | | | |  | 5 | Fuori reparto | | | |  |  | 5 | Leggera/Libera | | | |  |
| 6 | Scale | | | |  |  |  |  | | | |  | 6 | Scale | | | |  |  |  |  | | | |  |
|  |  | | | |  |  |  |  | | | |  |  |  | | | |  |  |  |  | | | |  |
| Note: | | | | | | | | | | | | | | | | | | | | | | | | | |


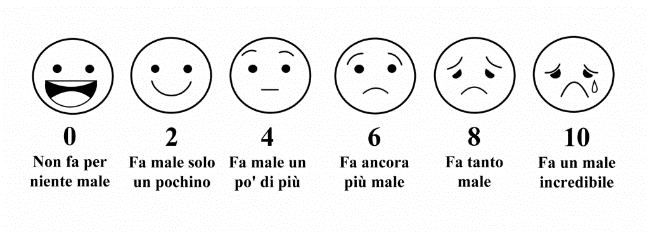

Supplement: Supplementary file 1 [file Supplementaryfile1.docx]
